# Worry About COVID-19 and Other Extreme Events Amongst Educators in Australia

**DOI:** 10.1177/00049441231168447

**Published:** 2023-04-26

**Authors:** Tamara Van Der Zant, Katherine L Dix

**Affiliations:** 1974 University of Queensland, Australia; 56366 Australian Council for Educational Research, Australia

**Keywords:** worry, wellbeing, educators, teachers, school, preschool, extreme events, COVID-19

## Abstract

The significant disruption of COVID-19 on schooling has heightened concerns about its impact on educators’ wellbeing. The current study examined how educators’ worry regarding the COVID-19 pandemic compared to their worry about other extreme events, such as natural disasters and critical incidents (a death or suicide of a child, young person, or colleague). Educators report that they were most worried about COVID-19. Educators working in preschools were more worried about COVID-19 and natural disasters than those in primary and secondary schools. However, worry regarding critical incidents increased with the age of students taught. Worry was influenced by socio-economic advantage (SEIFA), whereby educators working in higher SEIFA communities were less worried about natural disasters and critical incidents but shared similar levels of worry about COVID-19 as educators in lower SEIFA communities. With a better understanding about how different types of worry and levels of worry vary across different educator groups and different contexts, more effective supports can be developed and offered.

## Introduction

Amongst the many in-school factors that influence student learning outcomes and school engagement, educators^1^ are the most important (Hattie, 2015), and even more so in times of crisis. Yet, in efforts to stop the spread of COVID-19, governments around the world, including Australia, suspended entire school regions – estimated to affect more than 90% of the global student population (UNESCO, 2020). In Australia, many educators had to respond rapidly and adapt their teaching practice to ‘learning from home’. Bearing this enduring pressure, along with the rising cases, rising deaths, economic hardships, and widespread social and educational disruption over several years, is having adverse effects on the mental health and wellbeing of educators (Flack et al., 2021; Gore et al., 2020). Given the importance educators play in shaping the futures of young people, there is growing concern about the impact the COVID-19 pandemic is having on educator wellbeing (Allen et al., 2020; Dabrowski, 2021; McCallum, 2021). The increasing prevalence of symptoms of anxiety and worry during the pandemic have been reported in Australia (Dawel et al., 2020; Fisher et al., 2020; Fray et al., 2022; McCallum, 2021; Newby et al., 2020) and elsewhere (Allen et al., 2020; Pressley, 2021; Samuels et al., 2021).

Worry is an emotional response to an actual or potential threat and the associated uncertainty of the negative impact of the threat. Worry is typically characterised by repetitive thought patterns that focus on managing and avoiding the perceived threat (Borkovec et al., 1983; Lee & Hawkins, 2016; Lewis et al., 2018; Zysberg & Zisberg, 2020). As a concept, worry is closely associated with anxiety, stress and fear (Borkovec et al., 1983; Zebb & Beck, 1998; Zysberg & Zisberg, 2020).

Assessing worry is extremely pertinent in the context of the COVID-19 pandemic – a threat associated with great uncertainty and perceived helplessness – to estimate the additional relative-burden it may be taking on society. As early as February 2020, 20% of a representative sample of over 6000 people living in the United Kingdom reported being ‘very’ or ‘extremely’ worried about the COVID-19 pandemic (Smith et al., 2022). An international study involving 48 countries, conducted between March and May 2020, found that 72% of respondents reported feeling worried about the COVID-19 pandemic, and 65% anticipated further worry going forward, despite only 16% believing they were likely susceptible to contracting COVID-19 (Gamonal-Limcaoco et al., 2021). Taylor et al. (2020) found that worry about the COVID-19 pandemic was positively associated with anxiety, depression, health anxiety and obsessive-compulsive symptoms reported by American and Canadian adults.

Evidence is emerging that the disruption of COVID-19 on schools in Australia and abroad is negatively impacting teacher morale and wellbeing (Alves et al., 2021; Fray et al., 2022; Hascher et al., 2021; Kraft et al., 2021). However, educators are regularly called upon to deal with crises and are expected to manage the implications of crises that affect their learning community.^2^ Typical crises can include natural disasters and critical incidents, like suicide within a community. For example, Australian communities are enduring substantial damage from natural disasters and impacts are projected to increase in coming years (SGS Economics and Planning, 2016). With climate change and the environment rated as one of the greatest concerns for Australians (Australian Psychological Society, 2015; Patrick et al., 2021), those adversely impacted by natural events can continue to suffer poor mental health outcomes years later (McFarlane & Van Hooff, 2018; Reifels et al., 2017).

In addition to the COVID-19 pandemic and natural disasters, educators are also expected to manage localised critical incidents like the death of a child, young person or member of staff within a learning community. This can be an extremely distressing experience for educators, particularly in cases of suicide, and when compounded by having to manage and respond to the emotional needs of students and the wider community. Kõlves et al. (2017) reported that Australian teachers frequently felt inadquately equipped in the wake of such incidents. Given that suicide is the leading causes of death for young people in Australia, government-funded initiatives that provide suicide postvention support have become necessary (Be You, 2020; Graham & Truscott, 2017; Sartore et al., 2019). The additional burden of worry associated with the COVID-19 pandemic has not been studied against the worry caused by other community-level extreme events, such as bushfire, flood, and critical incidences – contexts that many educators also have to co-manage on an increasingly regular basis.

Relatively little has been reported about the demographic distribution of COVID-19 related worry (Samuels et al., 2021) and other extreme events. For example, socio-economic advantage has been associated with differences in outcomes following natural disasters (Cutter et al., 2003; Rufat et al., 2015). Because of its vastness, Australia has substantial variation in the risk of natural disaster across regions (SGS Economics and Planning, 2016). Demographic differences in educational communities are also likely to influence capacity to respond and recover from critical incidents. For example, child mortality is generally substantially higher in adolescents than primary school aged children and is lower in communities with greater socio-economic advantage (Australian Institute of Health and Welfare [AIHW], 2021b; Henley & Harrison, 2019). As with natural disasters and critical incidents, the impact of the COVID-19 pandemic was not experienced evenly across Australia (Flack et al., 2020). Substantial differences in rates of infection and restrictions to limit the spread of the disease existed between states, with Victoria recording nine times as many deaths by 29 April 2021 than the remaining states combined, despite representing approximately 26% of the Australian population (Department of Health, 2021). Given that the risk of different types of extreme event is broadly associated with demographic variation, it is likely that the worry experienced by educators regarding these events may similarly vary in association with the context of their learning community.

Better understanding the level and nature of worry that educators are carrying is important. The lack of extant literature suggests that studies are needed to investigate the distribution of COVID-19 worry compounded with other worries in the population generally, and in Australia’s education workforce in particular (Fray et al., 2022). By identifying groups in the community that may be most burdened by worry, appropriate strategies and programs can be implemented that support the mental health and wellbeing of educators under the greatest pressure.

Accordingly, in the current study, we present a simple scale that assesses worry across a number of extreme events to investigate what teaching contexts are associated with variation in worry regarding virus pandemics like COVID-19, natural disasters (i.e. bushfires, extreme weather like floods, storms and hail, or cyclones), and critical incidents (such as a death or suicide of a child, young person or colleague). Specifically, we address the following research questions:

*How does educators’ worry vary with school community socio-economic advantage, school type, and state location, and are some educators more burdened by worry than others?*

## Methodology

### Research Context

**Figure 1.**
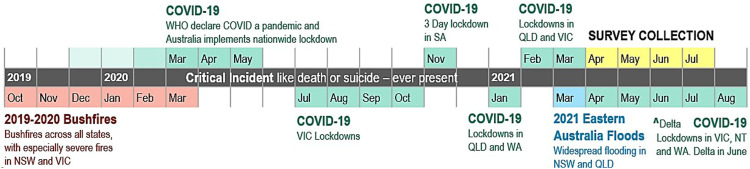
Timeline of recent worrying events in Australia

Studying the effects of COVID-19 on educators was not the main aim of the original research, which set out to evaluate the impact of two in-school wellbeing initiatives being rolled out across Australia prior to the pandemic – namely, the *Be You* national mental health in education initiative (Dix et al., 2022a) and the *Smiling Mind* primary school program (Dix et al., 2022b). In order to ensure that we were measuring the impact of the initiatives and not COVID-19 on educator outcomes, a simple moderator was developed to correct for the impact of COVID, if needed. The collection of evaluation data, timed during mid-2021, was well into the COVID-19 pandemic (see Supplement 2) but other events like the devastating 2019–2020 bushfire season (Black Summer), and widespread flooding in New South Wales and Queensland in March 2021, were still in the collective conscious (see Supplement 3). Figure 1 presents a brief timeline of significant events leading up to the data collection period that potentially heightened levels of worry in educators (Australian Bureau of Statistics, 2021a; COVID-19 Pandemic in Australia, 2023; List of Natural Disasters in Australia, 2023).

The devastating firestorms in late 2019 were still vivid in people’s minds with many communities directly impacted still rebuilding. This was followed by the emergence of the COVID-19 virus in Australia, which was declared a global pandemic by the World Health Organization in March 2020 (World Health Organization, 2020). While the following summer was thankfully less eventful, communities in eastern Australia endured torrential rains and flooding in March 2021. Through all of this, school communities continue to be impacted by critical incidences. The death rate of young people aged 15–19 in Australia is around 32 per 100,000 (AIHW, 2021b), with the leading cause being injury (intentional and unintentional). In 2020, 99 deaths by suicide occurred among children and adolescents (aged 5–17) with the majority (74%) occurring in those aged 15–17 (AIHW, 2021a). It was the occurrences of these types of adverse events and their potential to negatively impact educator wellbeing, that gave rise to the development of a brief measure of worry.

### A Brief Measure of Worry

While more comprehensive scales of COVID-19 related worry have been developed (e.g. the 11-item Coronavirus Impact Scale; Stoddard et al., 2021), this study used a brief purpose-designed measure, assessed on a five-point Likert scale (1 = *never*, 2 = *some of the time*, 3 = *half of the time*, 4 = *often* and 5 = *always*). Participants were asked:

*How often do you currently worry about…*

*a) Virus pandemics like COVID-19*

*b) Bushfire*

*c) Extreme weather like floods, storms and hail, or cyclones*

*d) A critical incident like death or suicide of a child, young person or colleague (included only in the Be You evaluation)*

Item reliability analysis (Cronbach’s α = 0.77) and confirmatory factor analysis (KMO = 0.80, *p* < 0.001) were employed and confirmed a single construct reflecting levels of worry. Possible scores on the instrument ranged from 1 to 5, with scores averaged to derive an overall indication of worry. A higher average score indicated a higher average level of worry.

### Participants

Questionnaires were administered to an existing cohort of educators^3^ as part of the independent evaluations of two unrelated wellbeing initiatives being implemented in Australian schools during 2021 (Be You and Smiling Mind). These self-selected cohorts of educators were similar to each other, with more respondents being part of the leadership team or focussed on leading wellbeing in the learning community. Moreover, participants represented learning communities that were demographically similar to the national profile of learning communities (see Table 1; Australian Bureau of Statistics, 2021e). Data used in this current analysis were included on the basis that participants had an active teaching role within their preschool or schooling context. Both evaluations were conducted by the same research team, affording the opportunity to include the same brief measure of worry in both questionnaires (Dix et al., 2022a, 2022b). Extracting the data of respondents from preschool or schooling contexts who participated in either questionnaire between April to July 2021, resulted in a pooled sample of 3210 educators in 1905 learning communities across Australia. The participants were mostly female (83.2%; 15.2% male; 1.6% other), which is typical of the demographic profile of educators in preschools and schools across Australia.

**Table 1.** Characteristics and representativeness of the educators and their learning communities in the pooled sample, compared to the national distribution of educators and sites in Australia.

Characteristic	Participants % (*n*)	Sites % (*n*)	Sites nationally %	Bias by site (S-N)
*N*	3210	1905	21958*	
State				
NSW	35.5% (1139)	34.1% (647)	32.4%	1.7%
VIC	21.2% (682)	21.9% (416)	25.6%	−3.7%
QLD	13.2% (423)	14.5% (277)	17.5%	−2.9%
WA	13.5% (432)	13.4% (255)	12.2%	1.2%
SA	7.4% (240)	8.6% (164)	7.0%	1.5%
TAS	2.3% (74)	2.6% (50)	1.9%	0.7%
ACT	5.7% (184)	3.5% (68)	1.9%	1.7%
NT	1.1% (36)	1.4% (28)	1.6%	−0.1%
SEIFA				
Low (decile: 1–3)	24.8% (795)	25.0% (476)	25.0%	0.0%
Medium (decile: 4–7)	36.2% (1163)	38.1% (725)	37.6%	0.4%
High (decile: 8–10)	39.0% (1252)	37.0% (704)	37.4%	−0.5%
Remoteness				
Metro	63.6% (2043)	65.2% (1169)	61.4%	−3.6%
Regional	32.0% (1028)	30.4% (642)	33.7%	3.1%
Remote	4.3% (139)	4.4% (94)	5.0%	0.5%
Institution type				
Preschool/kindergarten	9.0% (315)	12.5% (239)	30.2%	−17.7%
Primary	43.3% (1389)	44.8% (854)	41.3%	3.6%
Secondary	25.5% (819)	23.5% (448)	11.8%	11.7%
Combined	21.4% (687)	19.2% (365)	16.8%	2.4%

*Sites nationally are based on national estimate of preschools (Australian Bureau of Statistics, 2021d) and schools (Australian Bureau of Statistics, 2021b).

### Learning Community Demographic Measures

Table 1 presents the 3210 participants profiled by the demographic characteristics of their learning community. As this was a non-random self-selected sample, the demographic characteristics were collected as part of the evaluations to describe the cohorts and assess their representativeness against national distributions. The sample approximates representation (±5%) across state/territory, remoteness, and the learning community’s Socio-Economic Index For Area (SEIFA^4^), with over-representation of secondary schools and an under-representation of preschools.

### Statistical Analysis

To address the research questions, a Mixed Effects Model was fit to the data (GAMLj package in jamovi; Gallucci, 2020; see Supplement 1.). The dependent variable was level of worry on a 5-point scale from 1 (Never) to 5 (Always). The source of worry (COVID-19, bushfire, extreme weather, and critical incidents) was added as a fixed effect, along with the type of school (Preschool, Primary, Combined, and Secondary), States and Territories (ACT, NSW, NT, QLD, SA, TAS, VIC, WA), and SEIFA (Low, Medium, High). The interaction of the effect of source of worry with each of the other fixed effects was added. To account for variability associated within participant and within school or preschool, the intercepts of these factors were included as random factors. Holm-Bonferroni corrections were applied to follow-up analyses, and corrected *p*-values (*holm*) are listed where relevant.

## Results

### Model Fit

The mixed effects model (Akaike information criterion [*AIC*] = 29262.30) had a R^2^Marginal = .190 and a R^2^Conditional = .498. *R*^*2*^*Marginal* indicates the variability accounted for by the fixed effects within the model, and *R*^*2*^*Conditional* accounts for variability accounted for by fixed and random effects within the model. Therefore, the random effects, accounting for consistency of responding within participants and consistency of responding within schools, was more substantial than the combined effect of the fixed factors and interaction effects within the model. To further examine this effect, the Interclass Correlations (*ICC*) values for each random effect can be compared to determine which contributes most to explaining variability in the dependent variable. The *ICC* for School was .002, while the *ICC* for Participant was .380. This indicates that the participant as an individual, and not their context, influenced the level of worry reported. A Likelihood Ratio Test (LRT) further confirmed that while participant identity significantly predicted level of worry, *p* < .001, the specific community the participant was from did not, *p* = .911. Accordingly, participant identity was found to predict level of worry, which suggests the worries they experienced regarding each of the four event-types were potentially compounding and intercorrelated. In other words, if they were highly worried about one event, they were more likely to be worried about other events.

### Effect of Participant Identity on Worry

To examine the relationships between individual sources of worry, the correlation between different types of worry were calculated. Correlations between all types of worry were positive and statistically significant suggesting worry regarding each source was substantially related to worry on the others, *p*s < .001. Correlations between worry regarding different natural disasters (i.e. bushfire, extreme weather) was highest at *r* = .52. The remaining correlations ranged between *r* = .30 and *r* = .42 (see Table 2).

**Table 2.** Pearson’s *r* correlations.

	COVID-19	Critical incidents	Bushfire
Critical incidents	.381***		
Bushfire	.352***	.312***	
Extreme weather	.419***	.353***	.524***

*p < .05, **p < .005, ***p < .001.

### Source of Worry

**Figure 2.**
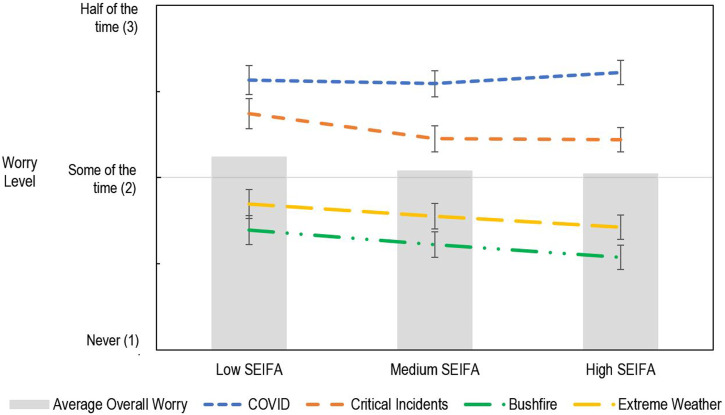
The effect of SEIFA on educators’ level of worry for each source of worry (SEIFA values mean centred)

The mixed effects model found the level of worry differed depending on which possible source of worry was being considered, *F* (3, 8858.01) = 340.08, *p* < .001. Educators reported the highest level of worry for virus pandemics like COVID-19, followed by critical incidents like death or suicide, then extreme weather and bushfire events, with all comparisons *p* < .001 (see Figure 2).

### Effect of Socio-Economic Background

Educators in socio-economically disadvantaged communities were significantly more worried, overall, compared to educators in more affluent communities, *F* (2,1335.76) = 5.10, *p* = .006. Specifically, educators working in communities with low-SEIFA status, *t* (1177.04) = 3.07, *p*_*holm*_ <.001 and *t* (1556.58) = 2.53, *p*_*holm*_ = .023 were more worried overall, than educators working in medium to high-SEIFA communities. There was no statistical difference in the overall levels of worry between educators working in medium and high-SEIFA communities, *t* (1201.64) = .65, *p*_*holm*_ = .52.

Additionally, the effect of SEIFA on level of worry differed for different sources of worry *F* (6, 8855.78) = 4.59, *p* < .001. Worry regarding bushfires, extreme weather and critical incidents was higher for educators working in low-SEIFA communities compared to high-SEIFA communities, *t* (3739.14) = 3.57, *p*_*holm*_ = .004, *t* (3751.97) = 3.04, *p*_*holm*_ = .022, and *t* (3900.83) = 3.32, *p*_*holm*_ = .010, respectively. Worry regarding critical incidents was lower for educators in medium-SEIFA learning communities compared to low-SEIFA communities, *t* (5083.20) = 3.26, *p*_*holm*_ = .001. Worry regarding COVID-19 did not differ across levels of SEIFA status, *p*_*holm*_*s* > .563 (see Figure 2).

### Effect of State Location

**Figure 3.**
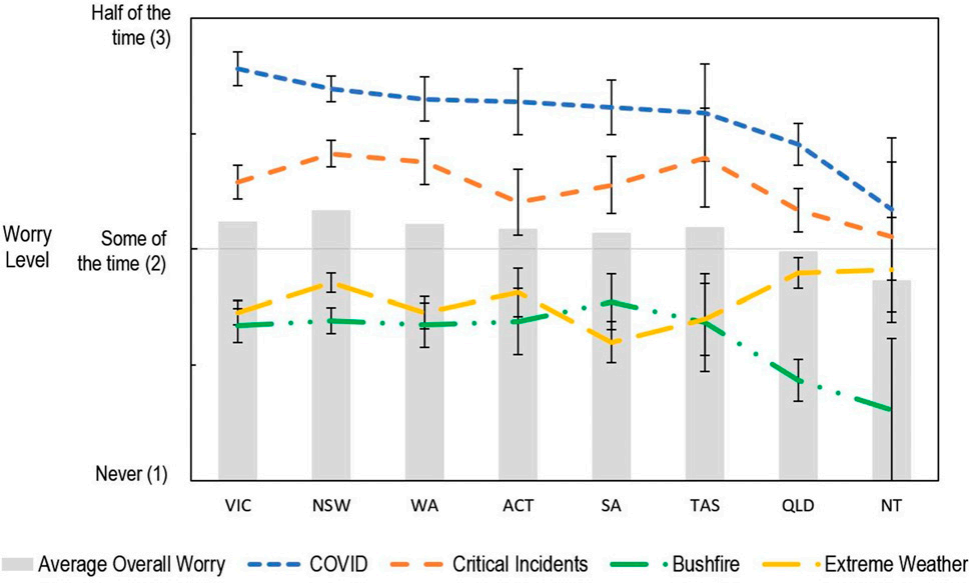
The effect of state/territory on educators’ level of worry for different sources of worry

The overall level of worry educators experienced varied across states and territories, *F* (7, 1016.97) = 3.76, *p* < .001 (see Figure 3 and Supplement 4). Educators in New South Wales reported higher levels of worry than educators in Queensland, *t* (836.27) = 4.49, *p*_*holm*_ < .001. No other comparisons were significant after correcting for multiple comparisons.

The extent educators worried about different adverse events also varied across states, *F* (21, 8855.17) = 6.27, *p* < .001. Worry regarding extreme weather like floods, storms and hail, or cyclones was higher in New South Wales and Queensland than in South Australia, *p*_*holm*_*s* = .012 and .007. Worry regarding bushfires was higher for educators in New South Wales, Victoria, Western Australia and South Australia than in Queensland, *p*_*holm*_*s* = .001–.028. Educators in New South Wales were more worried about critical incidents, like the death or suicide of a student or colleague, than educators in Queensland, *p*_*holm*_ = .001.

Educators in Victoria and New South Wales were more worried about Virus pandemics like COVID-19 than educators in Queensland, *p*_*holm*_*s* = .001. Educators in Victoria were also more worried about COVID-19 than educators in the Northern Territory, *p*_*holm*_ = .017. New South Wales and Victoria experienced the largest numbers of COVID-19 cases and deaths (by far) at the time educators were surveyed (see Table 3).

**Table 3.** Cases and deaths from COVID-19 across Australia’s states and territories as of 29 April 2021.

States/territories	Population	Cases of COVID-19	Cases per 100 000	Deaths from COVID-19
Victoria	6,649,200	20516	309	820
New South Wales	8,189,300	5464	67	54
Queensland	5,221,200	1556	30	7
Western Australia	2,681,600	998	37	9
South Australia	1,773,200	722	41	4
Tasmania	541,500	234	43	13
Northern territory	246,300	165	67	0
Australian capital territory	432,300	124	29	3

Cases of COVID-19 as of 29 April 2021 (Department of Health, 2021). Population estimates as of June 2021 (Australian Bureau of Statistics, 2021c).

### Effect of School Type

Educators’ overall level of worry was associated with the type of learning community they worked in, *F* (3, 1145.11) = 5.67, *p* < .001. Educators in preschools/kindergarten and secondary schools had higher levels of overall worry than those in primary schools, *t* (2402.57) = 3.53, *p*_*holm*_ = .003, and *t* (1114.83) = 2.99, *p*_*holm*_ = .014, respectively.

**Figure 4.**
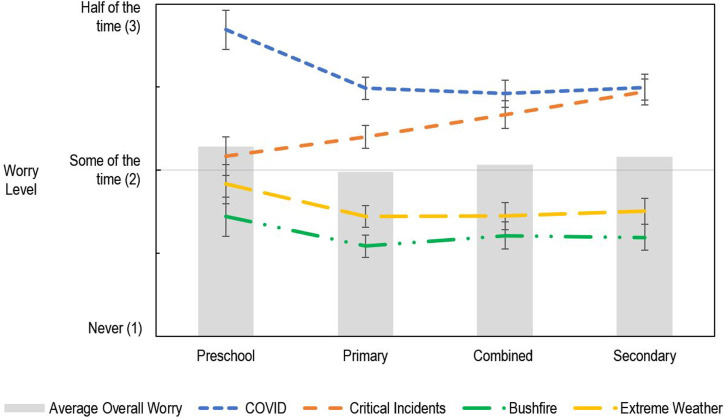
The effect of community type on educators’ level of worry for different sources of worry

The source of educators’ worry differed across educational level, *F* (9, 8853.38) = 15.33, *p* < .001. Educators in preschools/kindergartens were more worried than primary school teachers about bushfires, extreme weather, and COVID-19, *t* (7001.48) = 3.01, *p*_*holm*_ = .044, *t* (7061.29) = 3.29, *p*_*holm*_ = .018, and *t* (6988.39) = 5.96, *p*_*holm*_ < .001, respectively. Worry was also higher for educators in preschools/kindergartens compared to those in combined primary/secondary schools for extreme weather and virus pandemics like COVID-19, *t* (5223.23) = 2.98, *p*_*holm*_ = .044, and *t* (5132.74) = 5.95, *p*_*holm*_ < .001. Finally, preschool/kindergarten educators were also more worried about COVID-19 than secondary school teachers, *t* (6244.02) = 5.55, *p*_*holm*_ < .001 (see Figure 4).

With regard to critical incidents like the death or suicide of a child, young person or colleague, teachers in secondary schools and combined schools were more worried than those in primary schools or preschools/kindergartens, *t* (3638.78) = 6.48, *p*_*holm*_ < .001, *t* (6192.48) = 6.21, *p*_*holm*_ < .001, *t* (2837.79) = 2.99, *p*_*holm*_ = .044, and *t* (5142.22) = 3.87, *p*_*holm*_ = .002 (see Figure 4).

## Discussion

This study sought to explore whether features of school communities, such as socio-economic background, school type or state location were associated with variation in the level of worry teachers experienced. Secondly, we investigated whether, broadly, some educators were more burdened with worry than others. Participants reported their worry with regard to four possible sources of worry. Virus pandemics like COVID-19 was the topic participants reported worrying about most, regardless of location or socio-economic advantage. The second highest source of worry was in relation to critical incidents, like death or suicide. Extreme weather or bushfires events were associated with the lowest levels (though still substantial) of worry for educators (at a national level).

Worry about COVID-19, natural disasters or critical incidents, appeared to be related to a general propensity for worry. Educators who were more worried about one event were likely to be more worried about all topics, and vice versa. Moreover, those with generally high levels of worry or high propensity for worry about natural disasters or critical incidents, may have been more vulnerable to experiencing higher levels worry about COVID-19. Some evidence suggests an elevation of symptoms of mental illness during the pandemic has been particularly pronounced with regard to anxiety symptomology, for which worry is a prominent feature of measures and diagnoses (Asmundson et al., 2020; Kwong et al., 2021). The results may also reflect the compounding nature of worry that can lead to people feeling overwhelmed when faced with uncertainty and adversity on multiple fronts in their lives.

### Effect of Socio-Economic Background

Generally, there was association between educators’ worry, the socio-economic background of their learning community, and their expected vulnerability to each of the potential extreme events. For example, educators from socio-economically disadvantaged communities were more worried overall and reported higher levels of worry regarding critical incidents and natural disasters. Accordingly, critical incidents and natural disasters all present greater threat to socio-economically disadvantaged communities that are already under-resourced. Vulnerability to and recovery from natural disasters appears to be associated with socio-economic factors, with higher socio-economic advantage allowing people to absorb losses and access support more readily (Cutter et al., 2003; Rufat et al., 2015). Life expectancy and avoidable deaths, including disease, injury and suicide, are associated with socio-economic advantage (Afshar et al., 2020; Henley & Harrison, 2019; Korda et al., 2020; Welsh et al., 2021). Evidence also suggests that the relationship between socio-economic disadvantage and death from injury may be more pronounced among children (Henley & Harrison, 2019) and young people (AIHW, 2021b). Accordingly, our finding, that educators in lower socio-economic communities are more worried about critical incidents and natural disasters, appears to be calibrated to the level of risk their learning community experiences.

Despite experiencing more worry generally, communities with different levels of socio-economic advantage did not differ in the extent to which their educators were worried about virus pandemics like COVID-19. Evidence is emerging regarding how socio-economic advantage and disadvantage may relate to risk of negative experiences associated with the COVID-19 pandemic. As socio-economic advantage is associated with broad health outcomes, it is unsurprising that socio-economic disadvantage has been found to be associated with greater risk of acquiring and dying from COVID-19 (Bambra et al., 2020; Harris, 2020). Additionally, early evidence has shown that the largest negative impacts of lockdowns have typically been experienced by people with lower socio-economic advantage (Bower et al., 2021; O’Sullivan et al., 2020; Roder et al., 2022; Wright et al., 2020). As such, our initial finding (that worry about COVID-19 was not influenced by socio-economic advantage) appears to contradict the literature – that the worry educators’ report is not calibrated to the risk their community experiences. However, SEIFA, as a measure of socio-economic advantage and disadvantage, varies substantially with geography, whereby SEIFA scores typically decrease with remoteness. Research examining early COVID-19 transmission in the state of Victoria found that COVID-19 transmission was higher in metropolitan than regional areas (Roder et al., 2022). Therefore, while the risk of adverse outcomes from COVID-19 were likely lower for those experiencing higher socio-economic advantage, the areas with higher socio-economic advantage typically experienced higher rates of transmission. The lack of difference in worry between educators from communities in regions with different socio-economic advantage may be due to different but commensurate risk across the spectrum of socio-economic advantage at the point in the pandemic that this research was conducted. In other words, worry induced by the higher risk of contracting COVID-19 in highly populated areas like a city, was tempered by generally higher socio-economic advantage and greater access to resources to manage the impact of the disease.

### Effect of State Location

The impact of natural disasters is not evenly distributed across states and territories (SGS Economics and Planning, 2016; see Supplement 3). Measuring impact in insurance losses caused by extreme weather (including tropical cyclones, floods, storm and hail) the greatest impact was experienced in Queensland, closely followed by New South Wales, with the lowest impact suffered by the Australian Capital Territory, South Australia, and Tasmania (SGS Economics and Planning, 2016). The greatest impact of bushfires was experienced by Victoria, followed by New South Wales, with the lowest impact experienced by Queensland and Northern Territory (SGS Economics and Planning, 2016). This suggests that worry regarding natural disasters is roughly calibrated to the risk experienced in each state. With continued climate change causing increased severity and frequency of extreme weather, the impact of natural disasters, and the corresponding worry, is likely to increase (Royal Commission into National Natural Disaster Arrangements, 2020).

Child mortality, which may provide an approximate indication of the frequency of critical incidents involving the death or suicide of a student,^5^ is highest in the Northern Territory, followed by the Australian Capital Territory, and then by Queensland (Queensland Family & Child Commission, 2018). It is not apparent that the educators in New South Wales experience any greater risk of critical incidents than those in Queensland, with the available evidence suggesting the opposite to be more likely. It is also unclear as to why New South Wales educators may experience elevated worry in general, compared to Queensland educators. Further research is necessary to determine if these results are replicable, and if so, what may be leading to this elevated worry or what may be mitigating worry amongst Queensland educators.

Worry regarding virus pandemics like COVID-19 was greater for educators in Victoria and New South Wales than for educators in Queensland. Worry regarding COVID-19 was also greater for educators in Victoria than educators in the Northern Territory. New South Wales and Victoria experienced a much higher rate of COVID-19 cases and deaths than the other Australian states and territories (by far) at the time educators were surveyed (Department of Health, 2021). Though Queensland had experienced the third highest number of COVID-19 cases (being the third largest state), it had experienced the second lowest rate of cases relative to population, compared to the other states and territories (Department of Health, 2021). Similarly, though the Northern Territory had a high number of cases relative to the population, of the cases attributed to the Northern Territory, none had been due to community transmission within the territory (Northern Territory Government, 2021).

### Effect of School Type

Preschool educators reported experiencing high levels of worry across multiple topics. Little research has compared the level of worry Australian preschool, primary and secondary school teachers experience. The evidence available regarding the related construct of stress, however, has not found substantially higher levels of stress/worry in early childhood educators relative to primary and secondary school teachers (Carroll et al., 2022; Geng et al., 2015). An evaluation of teacher experiences across OECD nations found little difference in teachers’ work-related stress across educational levels (OECD, 2019). Research conducted just prior to the COVID-19 pandemic with Australian teachers found higher levels of stress in primary school teachers than secondary school teachers but did not have an adequate sample of preschool educators to detect differences in their stress levels (Carroll et al., 2022).

While it is not apparent from prior research that preschool educators would experience higher amounts of worry, there are multiple possible explanations for these results. Firstly, research has primarily focussed on teachers’ work-related stress and worry, while this study asks about worry for extreme events that may impact teachers within and outside the workplace. Therefore, there may be gaps in the research regarding educators’ experience of worry and stress beyond the classroom related to predisposing factors (e.g. differences in socio-economic advantage). Socio-economic factors can substantially impact how much people may be vulnerable to and worry about the different extreme events assessed in this research. In addition to the gender pay-gap that exists in Australia, there are differences in pay depending on your role: the average pay for an early childhood teacher ($1488/week) is substantially lower than for primary ($1801/week) and secondary ($1914/week) teachers (Department of Education, 2022). While our analysis account for the socio-economic context of the region of each learning community, it has not accounted for variation in socio-economic status associated with individual educators or the difference in professional conditions between educators working at different educational levels. Moreover, the self-selected cohort of Australian educators, while broadly representative, may have resulted in findings that are not generalisable to the wider population.

Further research is needed to establish whether the differences in worry found between educators are influenced by the Year levels they teach, and whether they relate to broader differences in mental health and wellbeing, as well as what leads to the variation in outcomes between educators.

Levels of worry were also generally high among secondary school educators, particularly with regard to critical incidents, such as the death or suicide of a child, young person or colleague. While it is not apparent that the death or suicide of a colleague would be more or less likely for educators working with students at different stages of their education, there is a higher likelihood of experiencing the death or suicide of a student for teachers working with adolescent students (Australian Bureau of Statistics, 2021a; AIHW, 2022). Mortality in Australia is substantially less likely between the ages of 1–9 years old, than for students 10–19 years old (Australian Bureau of Statistics, 2021a).

Various studies show that, globally, teachers experience significant grief and distress following the death of a student, with substantial disruption to their personal and professional life (Hart & Garza, 2013; Kõlves et al., 2017; Lazenby, 2006; Levkovich & Duvshan, 2021). Our results found that teachers working with secondary school students reported, on average, being worried ‘some times’ to ‘half the time’ about critical incidents, with 22.5% reporting being ‘often’ or ‘always’ worried. This level of worry is statistically similar to the level of worry regarding virus pandemics like COVID-19. While worry regarding virus pandemics will hopefully subside as the current COVID-19 pandemic continues to resolve, the ongoing risk of critical incidents is unlikely to change. This chronically high and ongoing level of worry experienced by teachers, further highlights the need for resources and interventions that support teachers and school communities to respond to critical incidents. While major initiatives like *Smiling Mind* target remote and disadvantaged communities and aim for prevention, *Be You* also offers a national approach to providing postvention support following a critical incident. It is hoped that this support is authentically available to the most at-risk disadvantaged communities. It also raises the question about what planning and resources may benefit teachers to reduce their worry about critical incidences. While there is limited research about the experience of teachers following the death of a student or colleague, one study found 35% of teachers believed the training opportunities to manage student grief were inadequate (Rowling & Holland, 2010). Moreover, among Australian primary and secondary teachers who had experienced the death of a student from suicide, 27% reported needing more support – specifically in the areas of counselling, crisis planning and appropriate acknowledgement of the incident (Kõlves et al., 2017).

### Limitations

This study was able to assess worry experienced by educators across the Australian education system as a secondary analysis of data collected for the concurrent impact evaluations of two unrelated wellbeing initiatives. Though this provided an excellent opportunity to assess our research questions at a much greater scale than is often possible, limitations exist associated with repurposing the data to address new research questions post-collection. Ideally, additional measures of participants own demographics would reduce error and allow greater insight into the extent features of the educational community that may impact the level of worry teachers experience compared to their own demographics. For example, information regarding the educator’s personal level of socio-economic advantage would allow research to assert the extent to which worry is associated with their own vulnerability to the extreme events assessed, compared to the vulnerability of their students. Additionally, the current measure uses single items to assess worry regarding each type of extreme event as was necessary to limit burden on participants in completing a lengthy survey. However, future research may seek to use a multi-item measure to more reliably and comprehensively assess the experience of worry.

Though the self-selected sample of educators who participated in this research was large and broadly nationally representative, there was an under-representation of preschool educators and an over-representation of secondary school educators. While these deviations from nationally representative proportions should not have biased the analyses generally, since mixed effects models are able to account for variability in sample sizes, the estimates of worry for groups represented by smaller samples may be less precise. As preschool educators were found to experience very high levels of worry, more thorough examination of this population with larger samples may allow for greater precision in estimating their level of worry and understanding their experiences. Future research is needed to explore how features of the learning communities’ that educators are working in may influence their worry, both inside and outside the workplace, with more comprehensive and tailored measurement of worry. This is particularly pertinent with increasing shortages in the teaching workforce and declining perceptions of the teaching profession (Heffernan et al., 2019; Thomson & Hillman, 2020).

## Conclusion

In this study we explored the research question: *How does educators’ worry vary with school community socio-economic advantage, school type, and state location, and are some educators more burdened by worry than others?* We find educators’ worry varies with the socio-economic context of their learning community, school type, and state location. Specifically, educators in schools with lower socio-economic advantage, educators in preschools, and educators in New South Wales experienced elevated levels of overall worry. There was alignment in educators’ worry regarding natural disasters, critical incidents, and COVID-19 with the risk presented by these threats to themselves and their communities. This highlights that while these extreme events may be infrequent, the worry remains persistent. Clearly, post-interventions in the aftermath of extreme events are necessary to manage acute impact. However, there may also be a need for pre-interventions to better prepare educators to manage these events and their persistent concern about them in order to alleviate their worry and support their coping.

As a key component of anxiety and predictor of burnout, it is essential to understand the causes of educators’ worry and who may be most susceptible to high levels of worry. While most research has narrowly focussed on the experience of worry, stress and wellbeing specifically related to educators’ work, more research is needed regarding educators’ wellbeing beyond the classroom and the learning community. Overall wellbeing, including the worries educators may experience outside the classroom, will inevitably influence their experiences and practices inside the classroom. Of greatest concern is that for many educators, their level of worry about a critical incident happening in their community is comparable to and compounded with their level of worry about COVID-19. With this information, interventions and supports can be more effectively offered to educators, particularly those burdened by worry on multiple fronts.

## Supplemental Material

Supplemental Material - Worry About COVID-19 and Other Extreme Events Amongst Educators in Australia Click here for additional data file. Supplemental Material for Worry About COVID-19 and Other Extreme Events Amongst Educators in Australia by Tamara Van Der Zant and Katherine L Dix in Australian Journal of Education

## Notes

1. The term ‘educators’ is used, rather than ‘teachers’, to be inclusive of those working in school or in preschool/kindergarten contexts.

2. The term ‘learning community’ is used to be inclusive of school and preschool/kindergarten contexts.

3. Once a school agrees to implement the initiative schoolwide, educators within the learning community are encouraged to become registered users to access the professional learning modules and resources.

4. SEIFA provides an indication of the socio-economic advantage and disadvantage across Local Government Areas.

5. Instances of child mortality among school age children that would not be considered critical incidents would only occur if the child was not attending school. As the proportion of Australian children not enrolled in school is small, instances in which a child dies will almost always be considered a critical incident managed by a school.
