# Supplementary material for: Worry About COVID-19 and Other Extreme Events Amongst Educators in Australia
Source: Aust J Educ. 2023 Apr 26;67(2):143–62. doi: 10.1177/00049441231168447 (PMC10133864; doi:10.1177/00049441231168447)

Worry about COVID-19 and other extreme events amongst educators in Australia: Supplemental Materials

Tamara Van Der Zant t.vanderzant@uq.edu.au
University of Queensland

Katherine L Dix Katherine.Dix@acer.org
Australian Council for Educational Research

Contents

Supp 1. R Code for Analyses 1
Supp 2. COVID-19 cases by LGA in QLD, NSW, VIC and ACT (as of 3 July 2020) 2
Supp 3. Natural Peril Risk Level by LGA 3
Supp 4. Worry Maps 4

Supp 1. R Code for Analyses

```
gamlj::gamljMixed(
        formula = Worry ~ 1 + School Type + State + SEIFA + Source_of_Worry +
        Source_of_Worry:SEIFA + Source_of_Worry:State + Source_of_Worry:School Type+( 1 |
        School Identifier )+( 1 | Participant Identifier ),
        data = data,
        plotHAxis = State,
        plotSepLines = Source_of_Worry,
        plotDvScale = TRUE,
        plotError = "ci",
        postHoc = ~ `School Type` + State + SEIFA + Source_of_Worry + Source_of_Worry:SEIFA +
        Source_of_Worry:State + Source_of_Worry:`School Type`,
        eDesc = TRUE,
        postHocCorr = c("none", "holm"),
        lrtRandomEffects = TRUE)
```

Supp 2. COVID-19 cases by LGA in QLD, NSW, VIC and ACT (as of 3 July 2020)

COVID-19 Cases by LGA in QLD, NSW, VIC, and ACT (as of 3 July, 2020)

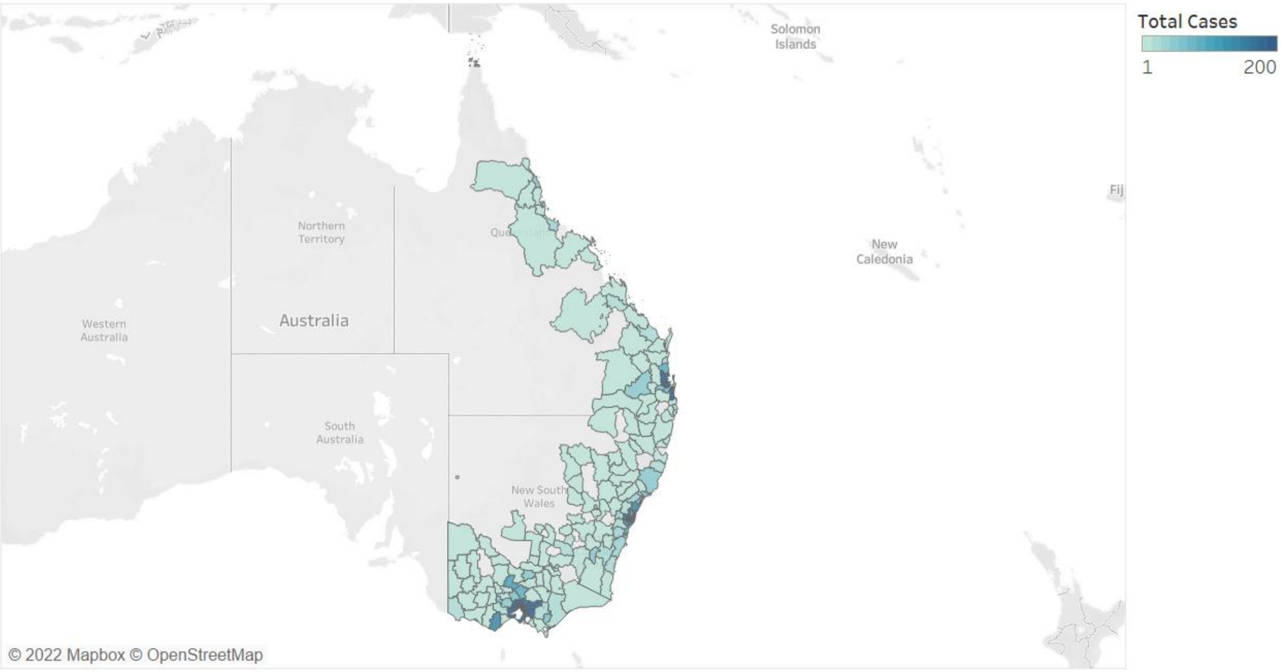

Map based on Longitude (generated) and Latitude (generated). Color shows sum of Total Cases. Details are shown for LGA.

Data used in figure sourced from:

NSW Ministry of Health. (2022). NSW COVID-19 cases by location. https://data.nsw.gov.au/data/dataset/aefcde60-3b0c-4bc0-9af1-6fe652944ec2

Queensland Government. (2022). Queensland COVID-19 Case Line List – Location & Source of Infection. https://www.data.qld.gov.au/dataset/7b90d88e-4f1f-4770-b721-5d91ca36c514/resource/1dbae506-d73c-4c19-b727-e8654b8be95a/download/opendata_qld_covidcase_loc.csv

State Government of Victoria (2022). Victorian COVID-19 data. https://www.coronavirus.vic.gov.au/victorian-coronavirus-covid-19-data

Supp 3. Natural Peril Risk Level by LGA

Bushfire risk by LGA

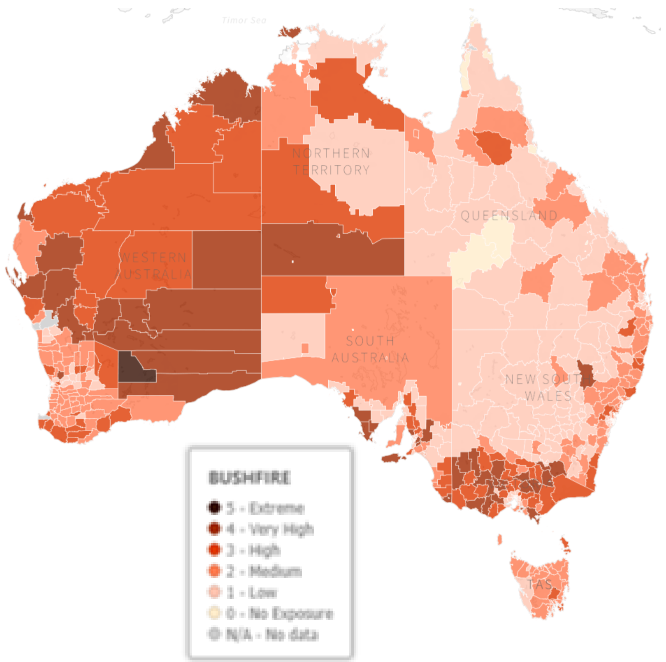

Tropical Cyclone (extreme weather) risk by LGA

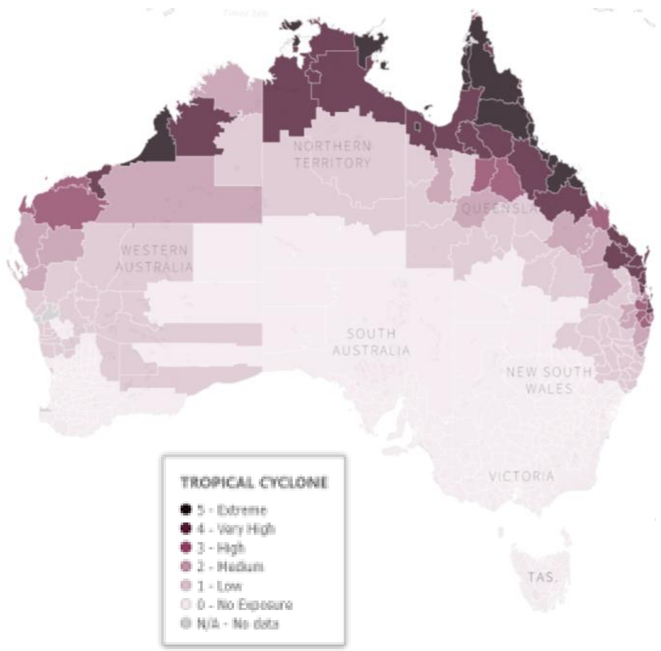

Flooding risk by LGA

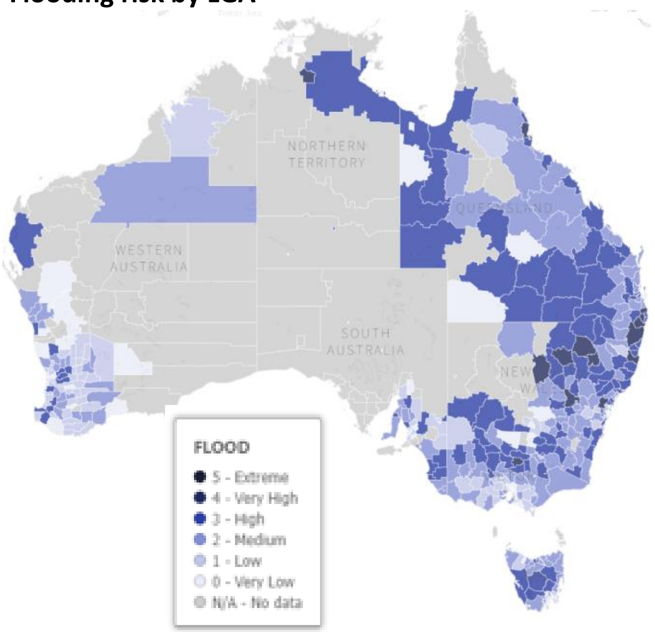

The maps provided here regarding risk of natural disasters have been sourced from SGS Economics and Planning. Natural Peril risk was measured using the Insurance Council of Australia's Low-resolution Exposure Address Dataset, or iLEAD. Natural Peril Risk is determined by past exposure to each form of natural peril. Risk bands are not comparable across maps.

SGS Economics and Planning. (2016). *At what cost? Mapping where natural perils impact on economic growth and communities*. https://www.sgsep.com.au/assets/main/SGS-Economics-and-Planning-at-what-cost-IAG-mapping-where-natural-perils.pdf

Supp 4. Worry Maps

Educators' worry for each cause across LGAs and states/territories based on 2021 questionnaire data

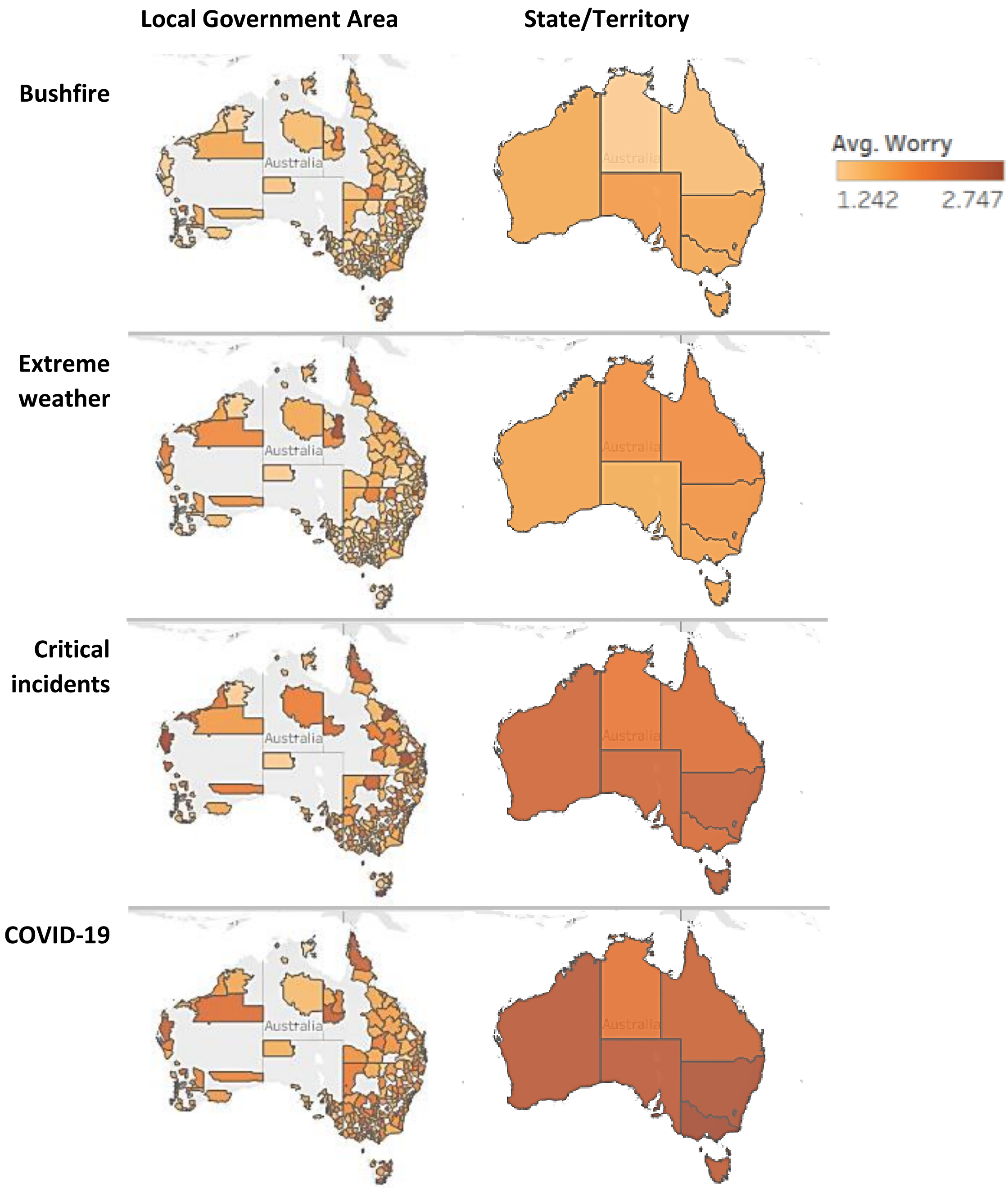

Supplement: Supplemental Material - Worry About COVID-19 and Other Extreme Events Amongst Educators in Australia [file sj-pdf-1-aed-10.1177_00049441231168447.pdf]
